# Effectiveness of Applying Green Heart, a Smartphone-Based Self-management Intervention to Control Smoking: A Randomized Clinical Trial

**DOI:** 10.34172/aim.2024.37

**Published:** 2024-05-01

**Authors:** Mojgan Ghavami, Alireza Abdshah, Ayat Ahmadi, Diba Akbarzadeh, Ali Mofidi, Mahnaz Ashoorkhani, Saeed Sadeghian

**Affiliations:** ^1^Cardiovascular Research Institute, Tehran Heart Center, Tehran University of Medical Sciences, Tehran, Iran; ^2^Department of Public Health Sciences, Miller School of Medicine, University of Miami, Miami, FL, USA; ^3^Knowledge Utilization Research Center, Tehran University of Medical Sciences, Tehran, Iran; ^4^Student Research Committee, School of Medicine, Shahid Beheshti University of Medical Sciences, Tehran, Iran; ^5^Department of Health Education and Promotion, School of Public Health, Tehran University of Medical Sciences, Tehran, Iran

**Keywords:** Mobile-health, Prevention, Smartphone, Smoking cessation, Technological interventions

## Abstract

**Background:**

Cardiovascular diseases (CVDs) pose a significant global health concern and are the most common cause of death and disability, necessitating preventive interventions targeting modifiable risk factors. Recently, mobile-health technology has been developed to improve the delivery of cardiovascular prevention by risk factor modification. The "Green Heart" mobile application (app) was designed to aid in risk factor control among coronary artery disease (CAD) patients.

**Methods:**

This parallel-group, single-blinded randomized controlled trial enrolled 1590 CAD patients, including 668 current smokers, randomly assigned to control (paper-based education) and intervention (application-based) groups. The app encompassed three modules targeting smoking cessation, dyslipidemia control, and blood pressure management. This study evaluated the impact of the smoking cessation module on behavioral change among current smokers. Green Heart assesses nicotine dependence, offering personalized quit plans, educational content, motivational messages, and automated progress tracking. The odds of smoking behavior changes during the 24-week follow-up underwent assessment.

**Results:**

The intention-to-treat analysis highlighted significantly elevated rates of smoking cessation and reductions in the intervention group versus the control group. Adherence to the app (per-treatment analysis) also demonstrated significantly more favorable smoking behavior changes among the application users. Logistic regression emphasized higher odds of quitting and reduction in smoking in the application group, showing an odds ratio of 2.14 (95% CI: 1.16–3.97) compared to those not using the app (*P*=0.015).

**Conclusion:**

Our results confirmed that complete adherence to the app for at least 24 weeks was linked to alterations in cigarette smoking behavior among CAD patients.

**Trial Registration Number:**

IRCT20221016056204N1

## Introduction

 Cardiovascular diseases (CVDs) encompass a range of conditions such as coronary heart disease, heart failure, stroke, and hypertension (HTN). These disorders are relatively common, with an overall prevalence of 48% among adults aged 20 years and older.^1^ In 2016, CVD accounted for 17.6 million deaths, reflecting a notable increase of 14.5% compared to 2006.^2^ Moreover, CVD stands as the leading cause of mortality worldwide, and projections indicate that it will be responsible for approximately 23.6 million deaths by 2030.^3^ CVDs, as the most common cause of death and disability in Iran, account for nearly half of annual mortality in Iranians.^4^

 To mitigate the risk of CVD, numerous studies have examined modifiable risk factors. The pioneering Framingham study paved the way for subsequent research endeavors aimed at identifying these potential risk factors. Framingham and other studies have consistently identified HTN, diabetes, obesity, hyperlipidemia, tobacco smoking, sedentary lifestyle, and inadequate physical activity as common risk factors that can be modified to prevent CVD.^5^ Three important studies have been performed to complete these findings. First, the INTERHEART study suggested current smoking and raised apolipoprotein (Apo) B/Apo A1 ratio as the two strongest risk factors for myocardial infarction, followed by a history of diabetes, HTN, and psychosocial factors.^6^ Furthermore, the INTERSTROKE study identified 10 potentially modifiable and prevalent risk factors that accounted for 90% of the population attributable risk for stroke. These factors encompassed a history of HTN or blood pressure of 140/90 mm Hg or higher, regular physical activity, Apo B/Apo A1 ratio, dietary patterns, waist-to-hip ratio, psychosocial factors, current smoking, cardiac causes, alcohol consumption, and diabetes mellitus.^7^ Lastly, the prospective cohort study known as PURE reported HTN as the most significant risk factor for CVD. This was followed by high non-high-density lipoprotein cholesterol levels, household air pollution, tobacco use, poor diet, low education, abdominal obesity, and diabetes.^8^

 Smoking is one of the most significant behavioral factors affecting human health and one of the most important causes of premature death.^9,10^ It has been known to be associated with changes ranging from low-grade inflammation to oxidant-antioxidant imbalance to cancer and many other chronic conditions. Although some of these changes have been observed to be reversible after discontinuation, others such as low-grade inflammation, persist for a long time, even after smoking cessation.^9^ Smoking cessation has been linked to greater life expectancy, particularly at younger ages; however, the benefits exist at all stages of life.^10,11^

 There are numerous methods of treatment available, ranging from nicotine-based to other medications and counseling.^10^

 Recently, newer technologies and smartphone-based methods have also been developed, which are relatively inexpensive, accessible, and convenient, with great effectiveness in improving the delivery of cardiovascular prevention and the ability to overcome communication barriers such as long distances.^12^

 The use of mobile-based interventions is effective in promoting smoking cessation while reducing tobacco use, at least in high-income countries.^13^

 Despite the diverse features of smoking cessation apps, gaps remain in their development, testing, and reporting, hindering optimal treatment reach and efficacy. Recent reviews have highlighted the increasing adoption of theoretical frameworks in app design, a positive step in the field.^14^ However, concerns exist about the suitability of existing theories for the evolving landscape of interactive Mobile-Health (mHealth) interventions.^15^ A recent literature review of the 55 articles systematically classified features of 33 apps that targeted smoking in general and specific populations. While this study identified a rise in early-phase app development studies, crucial details regarding this stage are often missing. According to this review, to maximize access to smoking cessation treatment and combat tobacco-related diseases, future research must prioritize the standardization and optimization of app development, testing, and reporting practices. This will not only refine treatment efficacy but also enhance transparency in the scientific process. This study underscores the need for more programmatic approaches to mobile app development in smoking cessation while welcoming the positive trend of increased early-phase research reports. Further efforts are needed to establish clear links between specific features and clinical outcomes. Additionally, although few apps have undergone rigorous large-scale clinical trials, consistent with earlier reviews, progress is evident in reporting transparency through protocol papers and clinical trial registration.^16^

 Considering that this technology is lacking in Iran, we developed the “Green Heart” application as an aid to smoking cessation and reduction of other cardiovascular risk factors. This study design intends to contribute to the existing literature on smoking cessation interventions and provide insights into the potential of mobile applications in promoting smoking cessation in patients with coronary artery disease (CAD). This clinical trial study aims to investigate the impact of a 24-week mobile application intervention on achieving smoking abstinence in patients with CAD and compare its effectiveness with routine care. Additionally, the study will examine the level of adherence to the application throughout the study period and its impact on quitting smoking.

## Materials and Methods

 This parallel-group single-blinded randomized controlled trial (RCT) study has been designed to evaluate the impact of using a Mobile-Health (mHealth) application on risk factor control.

 Individuals aged between 25 and 75 years who have documented CAD by coronary angiography in Tehran Heart Center and have at least one uncontrolled risk factor (HTN, dyslipidemia [DLP], and current cigarette smoking [CS]) from November 2022 to February 2023 were included in this study.

 Tehran Heart Center is a major academic tertiary-care hospital for cardiovascular disorders in Iran, which is affiliated with Tehran University of Medical Sciences.^17^

 We designed a mobile application (App), Green Heart, in 2022 in hopes of helping to reduce cardiovascular risk factors for secondary prevention in CAD patients. The application consists of three interventions to help quit smoking, control DLP, and aid in the management of blood pressure.

 The module of the app that targets smoking cessation includes baseline and follow-up questionnaire forms to track the status of the patient and assess the degree of nicotine dependence by the Fagerström test.^18^ The reliability data of a study that assessed the test-retest stability of the Fagerström test for nicotine dependence (FTND) confirmed that this test is highly reliable.^19^ Additionally, the study that evaluated the psychometric properties of the Persian version of the FTND for Iranian smokers demonstrated satisfactory results, and the instrument could be applied in tobacco control programs to assess nicotine dependence.^20^

 The app also asks questions about the duration of smoking, the frequency of quitting attempts, the daily cost of smoking, the use of hookah, the state of the desire to smoke, and the reason for the desire to smoke. This module also provides a stream of educational content on the risks of smoking, benefits of quitting, techniques, tips on how to cope with cravings and withdrawal, and a stream of supporting, motivational, encouraging, and warning messages (e.g., the effect of quitting on improving health and the amount of recovered cost in case of complete smoking cessation). Green Heart builds a personalized quit plan and displays automated messages and feedback to help the patient follow the plan. The National Institute for Care and Excellence smoking cessation guideline was incorporated into the algorithm for developing the app.^21^ Connecting to the internet is not needed during the process of answering questions and receiving advice and reminders. Whenever the user connects to the internet, the data are collected and saved to the server.

 The programming languages for designing web services, data management panels, and mobile software were PHP 8.1 and Kotlin 1.7, respectively.

**Figure 1:**
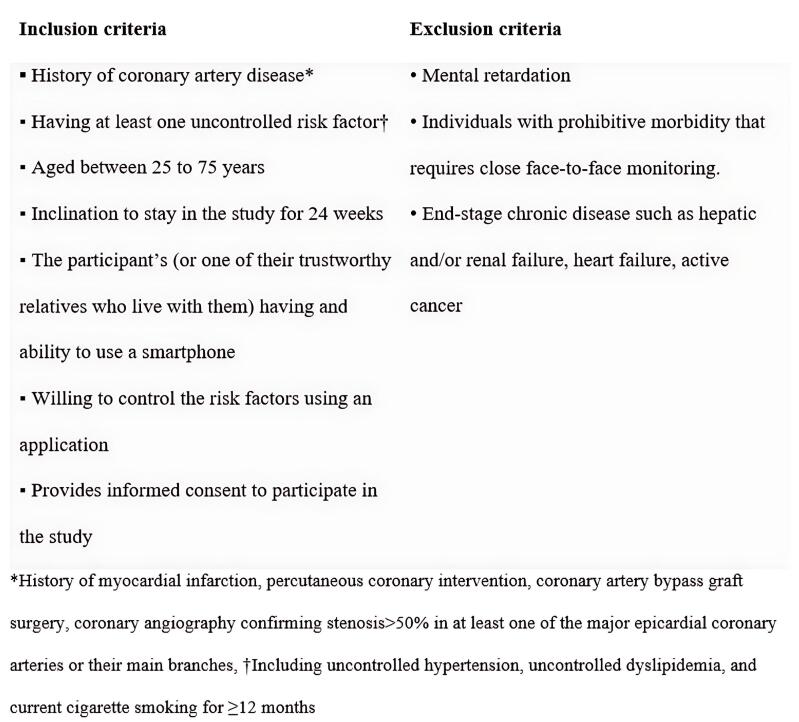
Eligibility Criteria for Participants

 Current smoking was defined as an average of ≥ 5 cigarettes daily use in at least the year before enrollment. Overall, 1590 eligible patients were enrolled, according to the criteria shown in Figure 1.

 To ensure balanced representation across a spectrum of risk factors, patients were randomly assigned to study arms using a stratified block randomization approach. This involves dividing participants into seven distinct groups based on the presence and combination of several key risk factors, as follows:

1. CS only
2. HTN only
3. DLP only
4. CS and HTN
5. CS and DLP
6. DLP and HTN
7. CS, HTN, and DLP

Within each group, randomization to the two control (conventional paper-based treatment: 795 subjects) and intervention (Green Heart app: 795 subjects) study arms was conducted with a 1:1 allocation rate utilizing variable block sizes (2, 4, 6, 8, and 10). A computer-generated random number list will be utilized using permuted block stratified randomization (Block stratified randomization Windows, Version 6.0, by Steven Piantadosi, M.D., Ph.D., Cedars-Sinai Medical Center).

**Figure 2:**
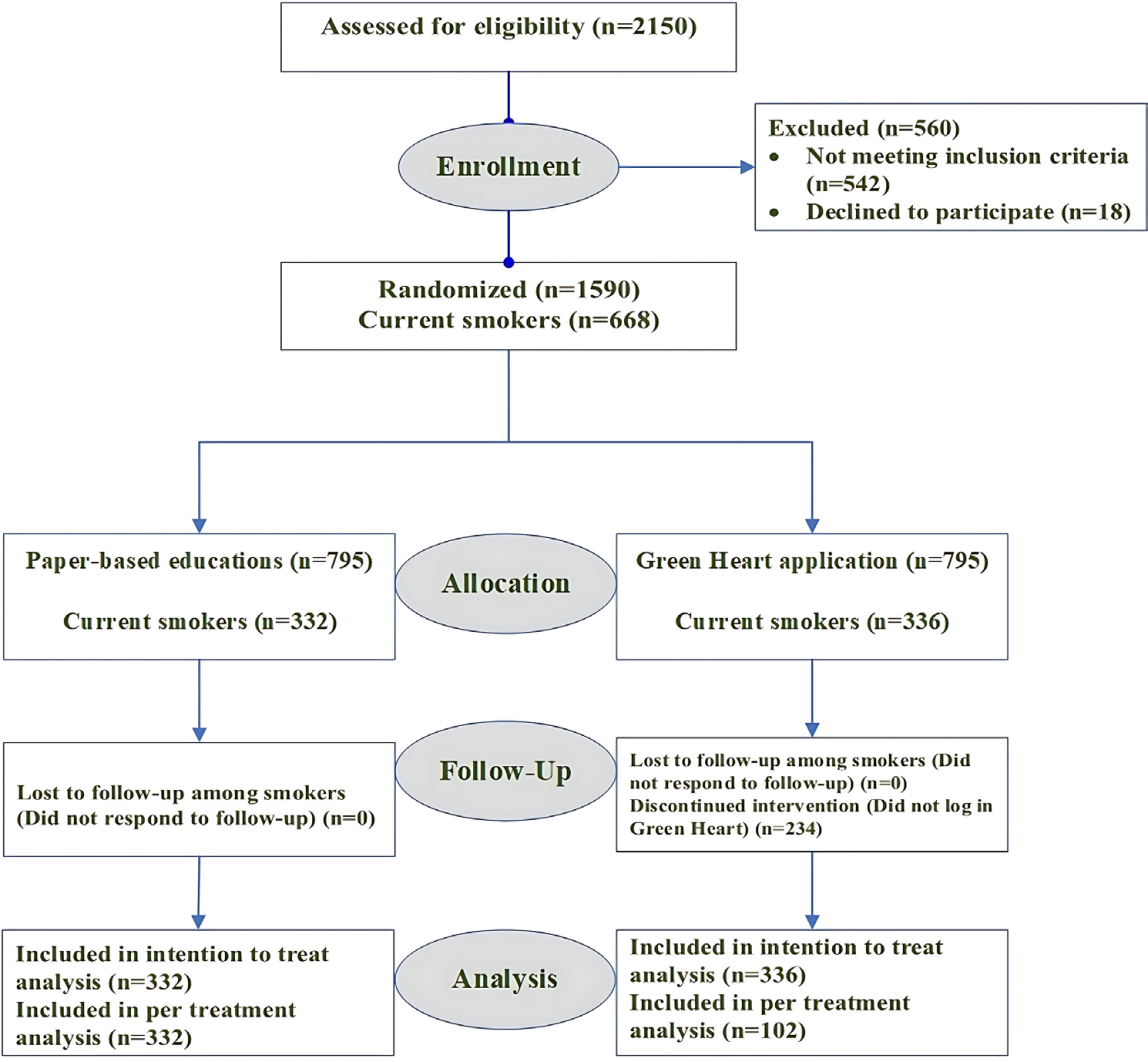
Consort Flow Diagram of the Green Heart Trial

 The practitioners who evaluated the risk factors controlling status were blinded to group assignment. Of the participants, 332 in the conventional group and 336 in the application group were current smokers. Details are depicted in Figure 2.

 The patients in the conventional group were supplied with pamphlets regarding the importance of smoking cessation in the prevention and management of CVDs.

 The software app was installed on the mobile devices of participants in the intervention group by our team. Participants and one of their trustworthy relatives who lived with them were also taught how to use the app. To resolve the problems, the patients were asked to work with Green Heart in the presence of our staff. To respond to any existing questions, they were given a telephone number for technical support. During the study period, patients in the intervention group were encouraged to continue to use the app.

 The patients were followed for 24 weeks, and the success of each treatment on smoking cessation or reduction in smoking was compared. Successful smoking cessation was considered continuous abstinence for at least 4 weeks during the 24-week follow-up. Logging “system-usage” data and the number of filled-out self-report questionnaires were recorded to measure adherence to the application.

 The primary and secondary outcomes were defined as successful smoking cessation and the number of participants who remained active users of the app till the end of the study, respectively.

 After 24 weeks, the status of risk factors, including smoking status, was evaluated through outpatient visits by a cardiologist for both arms. In case of an inability for participants to have a face-to-face visit, smoking status was asked by telephone call.

 The data were analyzed using R Studio and packages base R,^22^ tidyverse,^23^ and nnet.^24^ The chi-square test (or Fisher’s exact test where appropriate) and t-test (or Kruskal-Wallis as a non-parametric equivalent where appropriate) were used to compare the distribution of categorical and continuous variables, respectively. Univariable and multivariable binomial and multinomial logistic regression were utilized to estimate the odds of “smoking cessation” or “reduction in smoking” compared to “no change or increase in smoking” in those using the new app vs. conventional treatment. Considering the prevalence of treatment success in the study, the odds ratios and their 95% confidence intervals (CIs) were reported, and a *P* value of 0.05 was considered significant throughout this study.

## Results

###  Intention to Treat Analysis

 In our study, 668 people were analyzed, including 332 current smoker subjects in the conventional paper-based education group and 336 in the Green Heart app group (Figure 2). A summary of patients’ age, gender distribution, HTN, DLP, and diabetes mellitus is provided in Table 1, Section A. Although there were some statistical differences in the baseline characteristics, there were no clinically meaningful differences between the two groups.

**Table 1:** Demographics of Participants [Section A: By the Randomization Group (Intention to Treat Analysis) and Section B: By the Treatment Group (Per Treatment Analysis)]

		**Paper-based Education** **(n=332)**	**Green Heart Application** **(n=336)**	**Total** **(n=668)**	***P*** ** Value** **Test**
Section A: Intention to treat	Age				
	Mean (SD)	62.6 (7.51)	57.8 (8.82)	60.2 (8.53)	< 0.001 *t*-test
	Median [Min., Max.]	63.0 [39.0, 75.0]	58.0 [33.0, 75.0]	60.0 [33.0, 75.0]	
	Gender				
	Male	309 (93.1%)	320 (95.2%)	629 (94.2%)	0.304 Chi-square
	Female	23 (6.9%)	16 (4.8%)	39 (5.8%)	
	Hypertension				
	No	120 (36.1%)	111 (33.0%)	231 (34.6%)	0.445 Chi-square
	Yes	212 (63.9%)	225 (67.0%)	437 (65.4%)	
	Dyslipidemia				
	No	28 (8.4%)	11 (3.3%)	39 (5.8%)	0.007 Chi-square
	Yes	304 (91.6%)	325 (96.7%)	629 (94.2%)	
	Diabetes mellitus				
	No	215 (64.8%)	247 (73.5%)	462 (69.2%)	0.018 Chi-square
	Yes	117 (35.2%)	89 (26.5%)	206 (30.8%)	
		**Group A+Group B** **(n=566)**	**Group C** **(n=102)**	**Total** **(n=668)**	***P*** ** Value** **Test**
Section B: Per treatment	Age				
	Mean (SD)	61.1 (8.11)	55.2 (9.07)	60.2 (8.53)	< 0.001 *t*-test
	Median [Min., Max.]	61.0 [38.0, 75.0]	55.0 [33.0, 75.0]	60.0 [33.0, 75.0]	
	Sex				
	Male	537 (94.9%)	92 (90.2%)	629 (94.2%)	0.104 Chi-square
	Female	29 (5.1%)	10 (9.8%)	39 (5.8%)	
	Hypertension				
	No	187 (33.0%)	44 (43.1%)	231 (34.6%)	0.062 Chi-square
	Yes	379 (67.0%)	58 (56.9%)	437 (65.4%)	
	Dyslipidemia				
	No	36 (6.4%)	3 (2.9%)	39 (5.8%)	0.260 Chi-square
	Yes	530 (93.6%)	99 (97.1%)	629 (94.2%)	
	Diabetes mellitus				
	No	386 (68.2%)	76 (74.5%)	462 (69.2%)	0.248 Chi-square
	Yes	180 (31.8%)	26 (25.5%)	206 (30.8%)	

*Note*. SD: Standard deviation; Max.: Maximum; Min.: Minimum.

 After 24 weeks of treatment, in the conventional arm, 5.4% completely quit smoking, while 63.9% reduced smoking, and 30.7% either used the same number of cigarettes or increased their use. In the application group, 11% completely stopped smoking, 75.3% reduced their use, and 13.7% either used the same amount or increased. The details of smoking status after treatment (based on randomization) are listed in Table 2, Section A.

**Table 2:** Changes in Cigarette Smoking Behavior Among Each Intervention (Section A: Per Intention to Treat and Section B: Per Treatment)

	**Paper-Based Education** **(n=332)**	**Green Heart Application** **(n=336)**	**Total** **(n=668)**	***P*** ** Value** **Test**
Section A: Intention to treat				
Did not reduce or even increased smoking	102 (30.7%)	46 (13.7%)	148 (22.2%)	< 0.001 Chi-square
Reduced smoking	212 (63.9%)	253 (75.3%)	465 (69.6%)	
Quit smoking	18 (5.4%)	37 (11%)	55 (8.2%)	
Section B: Per treatment	**Group A+Group B** **(n=566)**	**Group C** **(n=102)**	**Total** **(n=668)**	***P*** ** Value** **Test**
Did not reduce or even increased smoking	135 (23.9%)	13 (12.7%)	148 (22.2%)	< 0.001 Chi-square
Reduced smoking	399 (70.5%)	66 (64.7%)	465 (69.6%)	
Quit smoking	32 (5.7%)	23 (22.5%)	55 (8.2%)	

 The odds of changes in smoking status were estimated using logistic regression (Table 3, Section A).

**Table 3:** Section A: Odds of Being in the Green Heart Application Group for Each Level of Reduction in Use and Section B: Odds of Adherence to the Green Heart Application for Each Level of Reduction in Use

		**Odds Ratio**	**95% CI**	***P*** ** Value**
Section A	Quiting smoking vs. using the same or increasing the use	4.56	2.35–8.85	< 0.001
	Reduced smoking vs. using the same or increasing the use	2.65	1.78–3.94	< 0.001
	Quiting vs. reduced smoking	1.72	0.953–3.13	0.072
	Cessation and reduction of smoking vs. use of the same or increased use	2.80	1.9–4.14	< 0.001
Section B	Quiting smoking vs. using the same or increasing the use	7.46	3.42–16.3	< 0.001
	Reduced smoking vs. using the same or increasing the use	1.72	0.92–3.22	0.089
	Quiting vs. reduced smoking	4.34	2.39–7.85	< 0.001
	Cessation and reduction of smoking vs. using the same or increasing the use	2.14	1.16–3.97	0.015

 The analysis revealed that those assigned to the Green Heart app group had 4.56 (95% CI = 2.35‒8.85, *P* < 0.001) times and 2.65 (95% CI = 1.78‒3.94, *P* < 0.001) times the odds of quitting smoking and smoking reduction (respectively) compared to those who used the same or increased their cigarette number.

 Based on the results (Table 3, Section A), those who installed the app had 2.8 times the odds of either completely stopping or reducing their smoking (Odds ratio (OR) = 2.8, 95% CI = 1.9–4.1, *P* < 0.001). Comparing complete cessation vs. reduction, it was noted that those who were assigned to the app group had 1.72 times the odds of complete cessation after 24 weeks; however, this association was not statistically significant (95% CI = 0.9‒3.1, *P* = 0.072).

 A multivariable analysis was performed on the odds of treatment success, adjusting for other independent factors in our study. The participants who were assigned to the Green Heart application had higher odds of smoking cessation and reduction compared to those who were assigned to traditional treatment (OR = 7.61 and 3.74, *P* < 0.001 and *P* < 0.001, respectively). This effect, along with the comparisons of other predictors, is described in Table 4, Section A.

**Table 4:** Multivariate Analysis (Section A: Multivariable Odds of Being Assigned to the Green Heart Application Group Based on Each Level of Reduction in Use and Independent Characteristics, and Section B: Multivariable Odds of Adherence to the Green Heart Application Based on Each Level of Reduction in Use and Independent Characteristics)

		**Odds Ratio**	**95% CI**	***P*** ** Value**
Section A	Quit smoking vs. use the same or increase the use	7.61	3.71–15.6	< 0.001
	Reduced smoking vs. using the same or increasing the use	3.74	2.43–5.75	< 0.001
	Quiting vs. reduced smoking	2.03	1.08–3.830	0.028
	Age	0.92	0.898–0.938	< 0.001
	Gender (Female vs. male)	0.76	0.378–1.540	0.451
	Hypertension	1.31	0.915–1.880	0.141
	Dyslipidemia	1.73	0.789–3.78	0.172
	Diabetes mellitus	0.66	0.459–0.953	0.027
Section B	Quiting smoking vs. using the same or increasing the use	11.8	5.08–27.3	< 0.001
	Reduced smoking vs. using the same or increasing the use	1.95	1.02–3.73	0.044
	Quiting vs. reduced smoking	6.04	3.1–11.8	< 0.001
	Age	0.91	0.882–0.934	< 0.001
	Gender (Female vs. male)	3.02	1.27–7.13	0.012
	Hypertension	0.61	0.379–0.982	0.042
	Dyslipidemia	1.82	0.489–6.79	0.371
	Diabetes mellitus	0.85	0.501–1.44	0.543

###  Per Treatment Analysis

 Next, the patients were analyzed based on their treatment adherence. Among the 336 people who installed the app, 102 adhered to treatment during the study and followed through with it.

 The characteristics of the patients who followed the treatment are listed in Table 1, Section B. The 3 groups of participants were defined as follows:

 Group A received paper-based education. Group B received the Green Heart app but did not adhere to the treatment, and Group C received the app and adhered to the treatment for 24 weeks.

 Among the 102 people who used the app, 23 (22.5%) completely quit smoking, 66 (64.7%) reduced their use, and 13 (12.7%) either used the same amount or increased their use. Among the 566 receiving the paper-based education and receiving the app with no complete adherence, 32 (5.7%) completely quit, 399 (70.5%) reduced their use, and 135 (23.9%) either used the same number of cigarettes or increased their use (*P* < 0.001). The details are presented in Table 2, Section B.

 Next, logistic regression was used to estimate the odds of a change in smoking status among the people who adhered to the Green Heart. Those who adhered to the app had 7.46 (95% CI = 3.42‒16.3,* P* < 0.001) and 1.72 (95% CI = 0.92‒3.22, *P* = 0.089) times the odds of complete smoking cessation and smoking reduction compared to those who used the same or increased numbers, respectively. Those who had complete adherence to Green Heart had 2.14 (95% CI = 1.16‒3.97, *P* = 0.015) times the odds of either smoking cessation or reduction. Those who completely adhered to the app had 4.34 (95% CI = 2.39‒7.85, *P* < 0.001) times the odds of quitting smoking in comparison to those who reduced smoking. The details are provided in Table 3, Section B.

 A multivariable analysis was also performed to adjust for the effect of other independent variables in our study. It was found that those with adherence to Green Heart application as part of their care were more likely to experience complete smoking cessation and reduction compared to those who did not stop smoking or increased their use (OR = 11.8 and 1.95, *P* < 0.001 and *P* = 0.044, respectively). The details of the multivariable analysis are summarized in Table 4, Section B.

 Among the 102 people who adhered to the application, we documented their filled-out self-report questionnaires, including nicotine dependence based on the Fagerström test, pack years of smoking, number of times they had tried quitting, and whether they used hookah. It was observed that they had smoked an average of 21.8 years (standard deviation [SD] = 13.2) and 17.9 (20.8) pack years of cigarettes. It was also noticed that they attempted to quit smoking with a range of 0‒30 times, and 9.8% (10 participants) also reported concomitantly using hookahs. About 38% of those who failed treatment were in the high and moderate nicotine dependence groups, while 27.3% of those who reduced use and 4.3% of those who completely quit were in the high and moderate dependence groups (*P* = 0.036). Moreover, participants who failed had a significantly higher pack-year history of smoking (23.1%) versus those who reduced smoking (20.6%) and those who completely quit (7.41%) (*P*< 0.001). The details of their nicotine dependence and smoking history are listed in Table 5.

**Table 5:** The Smoking History of Participants Adhered to the Green Heart Application

	**Quit Smoking** **(n=23)**	**Reduced Use ** **(n=66)**	**Failed (Using the Same or Increase in Use) (n=13)**	**Total (102)**	***P*** ** Value**
Nicotine dependence based on the Fagerström test					
High	0 (0%)	4 (6.1%)	1 (7.7%)	5 (4.9%)	0.036 Fisher’s exact
Moderate	1 (4.3%)	14 (21.2%)	4 (30.8%)	19 (18.6%)	
Low to moderate	8 (34.8%)	30 (45.5%)	6 (46.2%)	44 (43.1%)	
Low	14 (60.9%)	18 (27.3%)	2 (15.4%)	34 (33.3%)	
Years of smoking					
Mean (SD)	17.4 (16.8)	22.4 (11.4)	26.8 (13.1)	21.8 (13.2)	0.101 Kruskal-Wallis
Median [Min., Max.]	15.0 [1.00, 52.0]	23.5 [1.00, 50.0]	30.0 [1.00, 42.0]	23.5 [1.00, 52.0]	
Pack-years					
Mean (SD)	7.41 (9.86)	20.6 (23.3)	23.1 (15.9)	17.9 (20.8)	< 0.001 Kruskal-Wallis
Median [Min., Max.]	1.75 [0.0500, 33.0]	15.0 [0.250, 164]	16.5 [0.200, 45.0]	14.5 [0.0500, 164]	
Number of quit attempts					
Mean (SD)	4.17 (6.56)	2.92 (4.33)	3.46 (3.89)	3.27 (4.85)	0.863 Kruskal-Wallis
Median [Min., Max.]	2.00 [0, 30.0]	2.00 [0, 30.0]	2.00 [0, 10.0]	2.00 [0, 30.0]	
Use of hookah					
No	23 (100%)	59 (89.4%)	10 (76.9%)	92 (90.2%)	0.063 Fisher’s exact
Yes	0 (0%)	7 (10.6%)	3 (23.1%)	10 (9.8%)	

## Discussion

 The findings of our study provide evidence supporting the effectiveness of utilizing a mobile app for smoking cessation and reduction. Our data indicated that providing guidance and instruction on the installation of the Green Heart app (intention to treat analysis) was significantly associated with changes in CS behavior (OR = 2.8), a decrease in the number of cigarettes consumed (OR = 2.6), and successful smoking cessation (OR = 4.6). Furthermore, our results demonstrated that complete adherence to the Green Heart app for at least 24 weeks (per treatment analysis) was linked to alterations in CS behavior (OR = 2.1) and a higher likelihood of smoking cessation (OR = 7.5). However, the association with a reduction in smoking did not reach statistical significance (*P* = 0.09).

 The findings of our study align with current state-of-the-art methods. Another RCT investigating the efficacy of a smartphone app called “Quit with Us” reached a similar conclusion. The researchers found that using Quit with Us (intention to treat analysis) was significantly associated with a higher smoking abstinence rate compared to the control group, specifically among young adults (58.4% [80/137] vs. 30.9% [42/136], risk ratio [RR] = 1.89, 95% CI = 1.42‒2.52, *P* < 0.001].^25^

 A recent meta-analysis of RCTs examined the effectiveness of smartphone apps and text-messaging systems in smoking cessation. Their results demonstrated that, compared to minimal cessation support, short message service or app text messaging systems led to a significant increase in short-term (3 months) (log RR = 0.50, 95% CI = 0.25‒0.75; I^2^ = 0.72%) and long-term (6 months) abstinence (log RR = 0.77, 95% CI = 0.49‒1.04; I^2^ = 8.65%).^26^

 Other studies have explored different telemedicine or e-health techniques to promote smoking abstinence. A systematic review comparing telephone-based approaches with self-help materials revealed that abstinence rates were higher among individuals who received multiple sessions of proactive calls (RR = 1.38, 95% CI = 1.19‒1.61; 14 trials, 32,484 participants; I^2^ = 72%).^27^ Internet-based apps have also been used in smoking cessation, although there seems to be a shift in methodology from telephone- or internet-based approaches to smartphone apps.^26,28^ Studies utilizing interactive and personalized features in their smartphone apps have shown increased rates of quitting.^26^ Additionally, the content of the app seems to influence the outcomes. An RCT comparing an accept and commit therapy (ACT) smartphone app with a US clinical practice guidelines (USCPG) smartphone app demonstrated that the ACT app was more effective in smoking cessation than the USCPG app (28.2% [293 of 1040] vs. 21.1% [225 of 1067]; OR = 1.49, 95% CI = 1.22-1.83; *P*< .001). The ACT app encourages allowing urges of smoking to pass, while the USCPG app focuses on teaching how to avoid urges.^29^

 The use of biochemical verification (in contrast to self-report) for smoking abstinence is a topic of debate. Some studies suggest that incorporating biochemical verification into smoking cessation apps can enhance intervention effectiveness.^30^ However, others have reported high levels of agreement between biochemical verification and self-reporting.^31^ The Society for Research on Nicotine and Tobacco Subcommittee on Biochemical Verification has recommended that biochemical verification of smoking abstinence is not necessary for such studies.^32^ Consistent with this recommendation, biochemical verification was not employed in our study due to specific methodological challenges associated with remote implementation. These challenges include difficulties in identifying the individual who provides the sample, significant attenuation, and high costs compared to the anticipated low percentage of deviation from a high reach-low intensity intervention.^29^

 When examining mHealth approaches to smoking cessation, outcomes are not limited to abstinence rates. Some studies consider a reduction in CS as an outcome.^33,34^ In our study, we also included a reduction in cigarette consumption as an additional outcome. Furthermore, patient satisfaction is believed to be linked to compliance and persistence with e-health methods.^35^ Therefore, it is suggested that future studies consider satisfaction as another outcome when evaluating smoking cessation apps.

## Strengths and Limitations

 This study possesses several strengths. First, to the best of our knowledge, it is the first RCT in Iran to utilize new technologies and mHealth for smoking cessation, contributing to the advancement of research in this field. Additionally, the randomization process minimizes the risk of bias, ensuring that any observed effects are likely a result of the intervention.

 However, there are certain limitations to acknowledge. This study was conducted in a single center, which may limit the generalizability of the findings to larger, multi-center populations. The follow-up period of 24 weeks may be insufficient to determine long-term smoking abstinence. Longer follow-up with a larger sample size would enhance the reliability and generalizability of the results.

 Another limitation is that self-reported outcomes were used without biochemical verification of smoking cessation or reduction. As previously mentioned, self-reporting may be less reliable than biochemical verification.

 Out of 336 patients assigned to the Green Heart application group, only 102 completely adhered to the app during study time, resulting in a relatively high dropout rate, which could introduce bias into the study.

 Acknowledging these limitations allows for a comprehensive understanding of the study’s strengths and weaknesses and provides avenues for future research to address these limitations and further advance the field of mHealth and smoking cessation interventions.

## Conclusion

 The “Green Heart” mobile application is an effective tool for smoking cessation and reduction among individuals with CAD and uncontrolled cardiovascular risk factors. The results indicated that the application was associated with significant changes in CS behavior, including increased odds of smoking cessation and reductions in cigarette consumption.
